# Supplementary figures and images for: Assessment of the effect of therapy in a rat model of glioblastoma using [18F]FDG and [18F]FCho PET compared to contrast-enhanced MRI
Source: PLoS One. 2021 Mar 5;16(3):e0248193. doi: 10.1371/journal.pone.0248193 (PMC7935304; doi:10.1371/journal.pone.0248193)

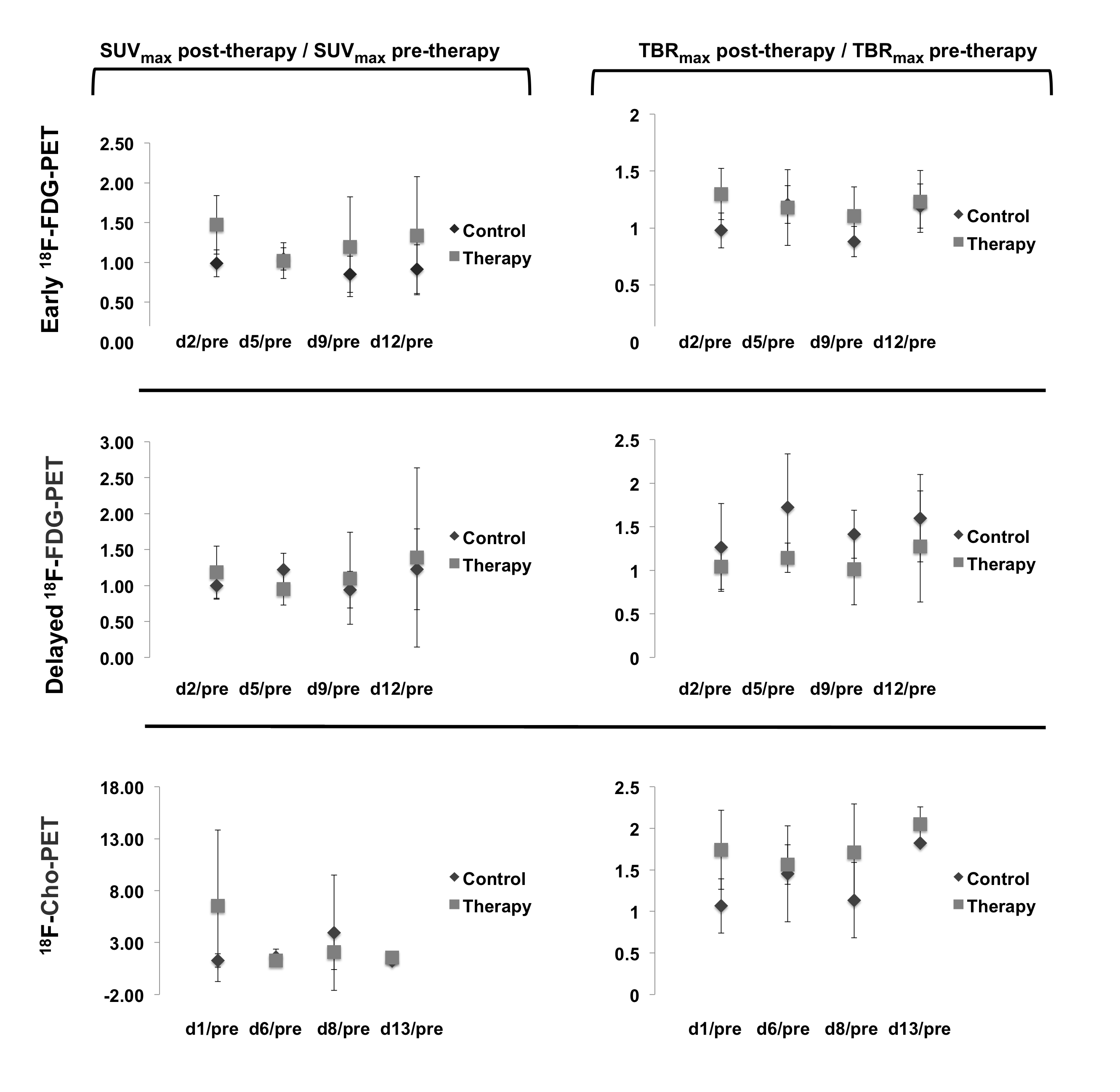

Supplement: S1 Fig — (TIF) [file pone.0248193.s001.tif]
